# Assessment of antibacterial activity and cytotoxic effects of *in vitro* and *in vivo* plant parts of a medicinal plant *Gynura procumbens* (Lour.) Merr.

**DOI:** 10.1016/j.heliyon.2023.e22954

**Published:** 2023-11-28

**Authors:** Sanchita Saha, G M Al Amin, Md Salim Khan, Barna Goswami, Farhana Afroz, Md Ahashan Habib, Shahina Akter, Tanjina Akhtar Banu

**Affiliations:** a Department of Botany, Jagannath University, Dhaka, 1100, Bangladesh; b Bangladesh Council of Scientific and Industrial Research (BCSIR), Dhaka, 1205, Bangladesh

**Keywords:** *In vitro* plant parts, *Gynura procumbens*, Antibacterial, MIC, Cytotoxicity

## Abstract

The goal of this study was to evaluate the antibacterial and cytotoxic effects of both the *in vitro* and *in vivo* plant part extracts of the medicinal plant *Gynura procumbens*. An effective protocol for regeneration and callus formation was developed using nodal segments and regenerated leaf explants, respectively. The highest fresh and dry weight calli were produced after four weeks of culture on Murashige and Skoog (MS) medium containing 2.0 mg/L BAP and 2.0 mg/L NAA, while the most shoots were produced on MS medium containing 1.0 mg/L BAP and 0.5 mg/L IAA. The *in vitro* shoots developed roots on MS media with 0.1 mg/L IBA. The antibacterial activity of extracts against various bacteria was examined to determine their significance (*p* < 0.05). The least significant difference (LSD) test results showed that the regenerated leaf extract had the highest antibacterial activity while the callus extract had the lowest. The minimum bactericidal concentration (MBC) and the minimum inhibitory concentration (MIC) were also established. Regenerated leaf extract had the highest toxicity and the lowest lethal concentration (LC50) value (1.21 ± 0.03 μg/mL) in a brine shrimp lethality bioassay. In contrast, callus extract had the lowest toxicity and the highest LC50 (11.09 ± 0.4 μg/mL). In addition, the *in vitro* cytotoxicity test results revealed that the callus and field leaf extracts had anti-cell-proliferative properties. The regenerated leaf and stem extract, however, could induce cell growth.

## 1 Introduction

*Gynura procumbens* (Lour.) Merr. is a medicinal shrub of the Asteraceae family. It has widespread habitats in Africa [1] and the tropics of Southeast Asia [2]. *G. procumbens* possesses a high therapeutic potential and is used as a traditional medicine for rashes, kidney problems, constipation, hypertension, migraines, diabetes, urinary infections, fever, dysentery, cancer, and inflammation [[3], [4], [5]]. Pharmacological studies have also revealed the potential of *G. procumbens* as a treatment for anti-hyperlipidemic, anti-herpes simplex virus, anti-cancer, and as an analgesic agent like others medicinal plants [[6], [7], [8], [9]]. Various medicinal plants viz. *Camelina sativa, Nigella sativa* have the significant benefit of displaying significant resistance to both cold and drought [10,11]. The bioactive compounds found in the leaves of *G. procumbens* have a number of positive health effects [12]. In this era of rising demand for herbal medicines, the medicinal properties of *G. procumbens* can be effectively used for the development of novel medications [3,13]. Cuttings, which are the traditional method of propagation for this plant, are unable to meet the plant's large-scale commercial demand, whereas the *in vitro* culture technique does so quickly, in a sterile environment, and without compromising quality [14]. Although the different biological functions of *in vitro* and *in vivo* plants can differ due to the variety of bioactive compounds, the biochemical properties of *G. procumbens* leaf have been well established by numerous studies, whereas the regenerated plant materials have not been fully explored [13,[15], [16], [17], [18], [19]].

The secondary metabolism of medicinal plants produces thousands of molecules with therapeutic potential [20]. There are many emerging medicinal plants with a wide range of phytochemicals, such as protein, fat, carbohydrate, fiber, alkaloids, fatty acids, glycosides, and polyphenols, that have great antioxidant potential, such as: [1,14,17,21,22]. In addition, many plant tissues and organs contain so-called endophytic organisms that confer resistance to biotic and abiotic stresses [21,23]. Biologically active compounds produced by medicinal plants allow them to inhibit the growth and virulence of various microbes [24]. Pathogenic bacteria have the genetic capacity to acquire and transmit drug resistance. Antibiotic resistance has emerged due to the extensive use of antibiotics in biomedical science, which negatively impacts public health [25]. Microbes resistant to multiple antibiotics have rapidly increased over the past few decades. It is now a worldwide crisis. In addition to antibacterial, bactericidal, and antioxidant properties, some unexplored or underexplored flora may also have antibacterial, bactericidal, and antioxidant properties [26]. The demand for antibacterial herbal drugs is increasing due to the acquired antibiotic resistance of different bacteria [27]. Also, the correct dose of medicinal plants must be figured out since a higher dose of bioactive compounds can harm a living body. For example, the bioassay of brine shrimp lethality can quickly and accurately tell if plant extracts are toxic to shrimp larvae *in vivo* [28]. On the other hand, the cytotoxic effect of plant extracts on human cells, such as cell viability and cellular growth, can be screened by an *in vitro* cell culture assay (ISO, 10993-5) [12].

Genetic variation among the regenerants may result from various factors, such as sterilizants, wounds, and high hormonal supplementation under *in vitro* culture conditions [29]. Evaluating biochemical constituents such as cytotoxicity, antibacterial activity, antioxidant activity, and genetic fidelity among regenerants compared to the mother plant is, therefore, necessary if the ultimate goal is to use the plant in pharmaceuticals and the study of genetic improvement. Unfortunately, the evaluation of the antibacterial potentiality and cytotoxicity of the field-grown micropropagated plants with the *in vitro*-regenerated plants of *G. procumbens* still needs to be adequately documented. Therefore, this research aimed to establish a protocol for the micropropagation of *Gynura procumbens* to have biological material for its reintroduction into the environment and obtain plant raw material for use in traditional medicine without affecting wild populations. As a result, the main focus was the comparative analysis of the antibacterial activities and cytotoxic effects of various mother plant parts and *in vitro* grown plant parts.

## 2 Materials and methods

### 2.1 *In vivo* plant materials

*Gynura procumbens* plant parts (leaves and stem) were taken from the Bangladesh Council for Scientific and Industrial Research (BCSIR) garden of medicinal plants in Dhaka, Bangladesh. The specimen was identified with the help of the Bangladesh National Herbarium, Dhaka, Bangladesh (Accession No. DACB 88050) and conserved at the BCSIR, Dhaka, for further study. After thoroughly rinsing them with distilled water, the collected plant materials were freeze**-**dried. The nodal segments were used as the explant for direct organogenesis. The healthy and fresh nodal segments and internodes were first surface-sterilized with tap water and mild detergent. The explants were then rinsed inside the laminar airflow cabinet with sterile distilled water after being soaked in 70 % alcohol for 1 min. In the last step, 0.1 % HgCl_2_ (w/v) was added while the mixture was shaken for less than a minute. Rinsing with sterile distilled water followed this. Then they were ready for inoculation on culture media for *in vitro* regeneration.

### 2.2 *In vitro* plant materials

Small pieces of sterilized nodal segments about 1.5–2.0 cm long were cut and put into a 250 mL conical flask with MS medium supplemented with 30 g/L sucrose added as a carbon source [30]. The media was augmented with various combinations and concentrations of plant growth regulators (PGRs), i.e., BAP (1.0–2.0 mg/L) alone or in combinations with NAA (0.1–1.0 mg/L) or IAA (0.1–1.0 mg/L) or 2, 4–D (0.1–1.0 mg/L). The pH of the medium was adjusted to 5.7 ± 1, and agar (Duchefa Biochemie B.V. The Netherlands) at 8 g/liter was used as a solidifying agent. The media were dispensed in culture vials, and these culture vials, each containing 20–25 mL of media, were autoclaved at 15 psi and 121 °C for 30 min for sterilization. The media was solidified in the vials before inoculation. All cultures were maintained in a growth chamber at 25 ± 2 °C, 65–70 % relative humidity, and a photoperiod of 16/8 h (light/dark) while receiving cool white fluorescent tube lights with 40 μmol/m^2^/s intensity. Regenerated shoots were subcultured to fresh medium at 21–25**-** day intervals for further multiplication. *In vitro* regenerated plantlets (45–48 days old) were harvested from the culture media. The regenerated leaves and shoots were collected separately. Half of the regenerated leaves were used as explants for callus culture. The other half of the regenerated leaves and regenerated stems were freeze-dried separately for antimicrobial and cytotoxicity tests.

### 2.3 Callus culture

For callus induction, *in vitro*-regenerated leaves were inoculated and cultured on MS media with different concentrations and combinations of 2,4-D (1.0–4.0 mg/L), BAP (1.0–4.0 mg/L) and NAA (0.5–4.0 mg/L) with 3 % (w/v) sucrose and 0.8 % (w/v) agar (Duchefa Biochemie B.V. The Netherlands). Cultures were incubated at 25 ± 2 °C under illumination in a 16 h photoperiod with 40 μmol/m2/s light intensity and a relative humidity of 70 %. Callus development was analyzed from the first week of inoculation of *in vitro* regenerated leaf explants up to three weeks. After two weeks of inoculation, callus induction frequency was recorded. Then, these were subcultured on fresh media, showing the best callus induction results in MS + BAP 2.0 mg/L + NAA 2.0 mg/L. Five replicates of the plant regeneration and callus induction frequencies were calculated, and each experiment was repeated at least three times.

### 2.4 Preparation of plants extracts

Extraction was done by the maceration method [31]. Freeze-dried plant materials (field-grown leaves, regenerated leaves and shoots, and callus) were ground separately into a fine, homogenous powder in a mortar and pestle. 20 g of each dried powdered plant material was soaked in 200 mL of 80 % methanol for 72 h under continuous agitation in a shaker at room temperature. Then they were filtered with Whatman filter paper no. 1 to attain a clear filtrate. The filtrates were evaporated and dried using a rotatory vacuum evaporator. The yields of extract were stored in small beakers at 4 °C.

### 2.5 Bacterial strains for antibacterial activity test

The microorganisms were obtained from the stock culture of the Microbiology Laboratory of the BCSIR, Dhaka. Five species of bacteria: *Escherichia coli*, *Salmonella typhimurium*, *Pseudomonas aeruginosa*, *Bacillus cereus*, and *Bacillus megaterium*, were used to assess the antibacterial potentialities of the plant part extracts. Among the five bacterial strains, two are Gram-positive (*B. megaterium* (ATCC 18), *B. cereus* (ATCC 14579)) and three are Gram-negative (*E. coli* (ATCC 8739), *S. typhimurium* (ATCC 13311) and *P. aeruginosa* (ATCC 27833)) bacteria.

### 2.6 Antibacterial activity of plant extracts

The agar-well diffusion method was used to assess the antibacterial activity of various plant materials [24]. Each plant material was prepared in three different concentrations for the test by dissolving in dimethyl sulfoxide (DMSO) 0.25 g/100 mL, 0.5 g/100 mL, and 1.0 g/100 mL, respectively. The freshly grown bacterial broth was spread out with a clean cotton bud on a Mueller-Hinton Broth (MHB) media plate. Four wells of 8.0 mm in diameter were made with the help of the cork borer. Three wells contained three different concentrations of extract (100 μl), and the fourth one contained DMSO (100 μl), which was used as the negative control. Ampicillin (10 μg) and Doxycycline (30 μg) were used as positive controls. The inhibition zone was measured on an mm scale after 18 h of incubation at 37 °C.

### 2.7 Determination of minimum inhibitory concentrations (MIC's) & minimum bactericidal concentration (MBC)

MIC is the minimum amount of extract needed to stop microbial growth after 24 h of incubation. The minimum bactericidal concentration (MBC) is the lowest amount of an antibacterial agent that will kill a particular type of bacteria [32]. The broth microdilution method determined the MIC and MBC according to the Clinical & Laboratory Standards Institute (CLSI) guidelines [33]. Serial dilutions of the plant material extracts were prepared using 10 % DMSO. MHB media prepared microorganism inoculums with a final concentration of 5 × 10^5^ CFU/mL in each well. 100 mL of plant extract was added to each well of the 96-well microplate. A total of fifty (50) mL of bacterial suspension was added to each well except the negative controls. Doxycycline and 10 % DMSO were used as the positive and negative controls, respectively. The plates were incubated at 37 ± 2 °C for 18–24 h. Antibacterial activity was evaluated by measuring absorbance at 690 nm wavelengths.

### 2.8 Brine shrimp cytotoxicity assay

The cytotoxic effects of *in vivo* and *in vitro* plant part extracts were evaluated by brine shrimp lethality bioassays. The eggs of brine shrimp were hatched for 24 h and screened to determine LC50 values against varying concentrations (200–0.39 μg/mL) of plant part extracts diluted in DMSO by the serial dilution method. Vincristine sulfate was used as the positive control, while DMSO was the negative control. Ten living nauplii of *Artemia salina* were transferred to each vial holding 5 mL of simulated seawater with the help of a Pasteur pipette.

### 2.9 *In vitro* cytotoxicity analysis

The cytotoxic potential of different *in vitro* and *in vivo* plant parts of *G. procumbens* was determined against human lung cancer cell lines (A549, CSL, Germany) by the Trypan Blue Exclusion Method [34]. Dissolving test samples in DMSO-produced stock solutions (1.0 mg/mL). Lung cancer cells were cultured in 75 cm^2^ sterile flasks on Modified Dulbecco's Eagles Medium (DMEM) supplemented with 1 % penicillin/streptomycin/neomycin (100 U/mL), (0.1 mg/mL), 10 % (v/v) fetal bovine serum (FBS), and 25 mM N-(2-Hydroxyethyl)piperazine-N′-(2-ethanesulfonic acid) (HEPES) in 5 % (v/v) CO_2_, pH 7.4 at 37 °C [35]. Cells were divided into three groups for each treatment, and the experiment was repeated three times. The comparisons were made among treatment groups and vehicle groups. Freshly prepared treatment doses were applied to 1-day-old T-25 cell culture flasks containing 2.5 × 10^6^ lung cancer cells. After 24 h of incubation, cells were harvested using trypsin. Counting was done for 1.0 mL of cell suspension with the help of an automated cell counter (LUNA-II™ Automated Cell Counter by Logos Biosystems, South Korea). Trypan blue revealed the number of viable cells. Two drops of cell suspension containing 0.4 % w/v trypan blue (1:1) were added to the surface of the hemocytometer and placed on an automated cell counter. The number of unstained (viable) and stained (non-viable) cells was counted. Cells were calculated as a percentage of dead cells by % of dead cells = [no. of dead cells/total number of cells] × 100.

### 2.10 Statistical analysis

The plant regeneration and callus induction frequencies were calculated for five replicates. A one-way ANOVA and graphical presentation were conducted using SPSS (version 22.0) and OriginPro 2018 software. All experiments for antibacterial activities were carried out in triplicates, and a two-way ANOVA was performed to examine the effect of extracts on bacteria using SPSS (version 22.0). The LSD test was carried out for multiple pairwise comparisons among treated samples and types of bacteria. For the analysis of the LC50 values and *in vitro* cytotoxicity results, continuous variables between groups were compared with a one-way analysis of variance (ANOVA) and a Post Hoc Tukey's test. The *p*-value of 0.05 was considered statistically significant.

## 3 Results

### 3.1 *In vitro* plant regeneration

**Fig. 1:**
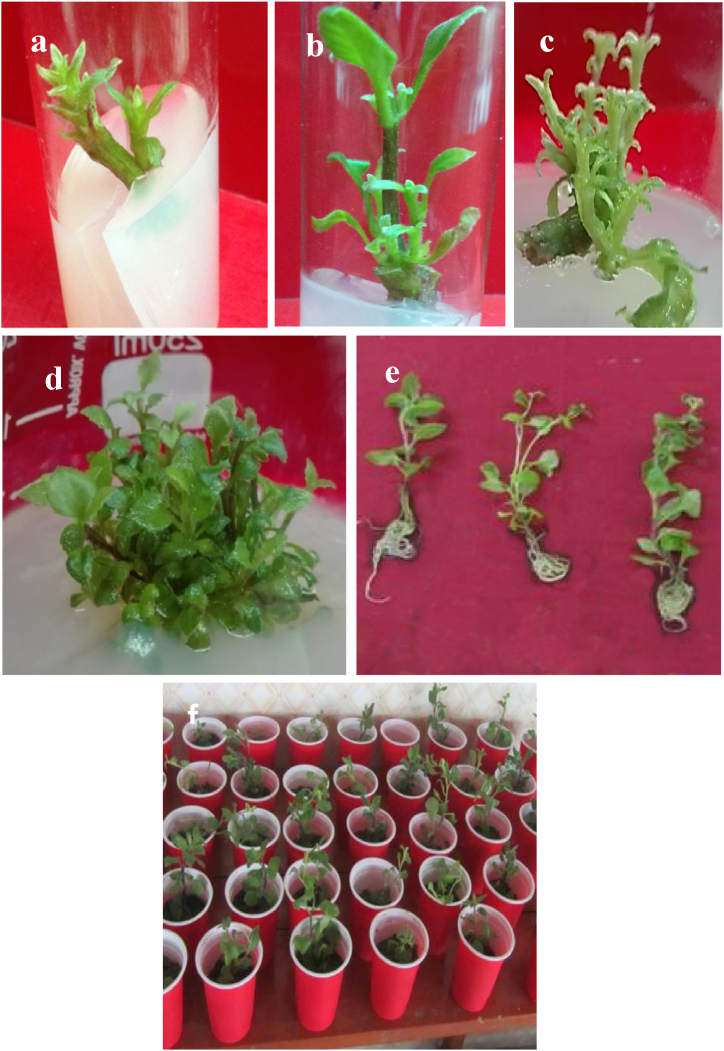
(a–f). Establishment of an *in vitro* regeneration protocol for *G. procumbens* using nodal explants. a) initiation of shoots on MS media supplemented with 1.0 mg/L BAP and 0.5 mg/L IAA; b) and c) elongation of multiple shoots in the same media as (a); d) showing multiple shoots; e) *in vitro* regenerated shoots showing roots on MS media supplemented with 0.1 mg/L IBA; f) *in vitro* regenerated plantlets hardening in pots.

The nodal segments of *G. procumbens* were cultured on MS medium supplemented with different concentrations of BAP (1.0–2.0 mg/L) either alone or in combinations with NAA (0.1–1.0 mg/L) or IAA (0.1–1.0 mg/L) or 2, 4-D (0.1–1.0 mg/L). The MS containing BAP 1.0 mg/L and IAA 0.5 mg/L showed the maximum number of multiple shoot proliferation, the best response (99.30 %), and the average shootlet length of 4.0–6.9 cm (Table 1). The mean number of shoots per explant was between 20.1 and 21.1 after 60 days of inoculation [Fig. 1 (a – f), Table 1]. *In vitro*, regenerated shoots were subcultured regularly on the same medium for further multiplication.

**Table 1:** Effects of different concentrations of PGRs on MS media on shoot regeneration from nodal explants of *Gynura procumbens*.

Plant growth regulator mg/L				% of responsive explants	Days required for initiation	Mean no. of shoots/buds (after 60 days)	Mean length of shoots (cm) (after 40 days)
BAP	NAA	IAA	2, 4-D				
1.0	**-**	**-**	**-**	58.20	5–8	6.0 ± 1.00	6.9 ± 1.05
1.0	1.0	**-**	**-**	43.00	6–10	5.6 ± 0.3	6.00 ± 0.01
1.0	0.5	**-**	**-**	75.50	5–7	8.9 ± 1.03	5.9 ± 0.75
1.0	0.1	**-**	**-**	83.80	5–10	6.3 ± 1.16	4.75 ± 1.03
1.0	**-**	**-**	1.0	49.00	6–10	5.3 ± 1.17	4.5 ± 1.16
1.0	**-**	**-**	0.5	54.20	5–10	5.0 ± 1.17	4.75 ± 0.3
1.0	**-**	**-**	0.1	61.50	5–7	4.9 ± 0.99	5.00 ± 0.01
1.0	**-**	1.0	**-**	73.00	4–8	7.23 ± 1.00	4.02 ± 0.75
**1.0**	**-**	**0.5**	**-**	**99.30**	4–7	**21.1 ± 0.2**	4.47 ± 0.01
1.0	**-**	0.1	**-**	69.00	4–10	10.33 ± 1.12	6.03 ± 0.75
2.0	**-**	**-**	**-**	74.00	4–8	11.33 ± 0.3	5.05 ± .0.33

Each value represents an average of 5 replicates and each experiment was repeated at least three times; values are expressed as Mean ± SD.

### 3.2 Callus culture

**Fig. 2:**
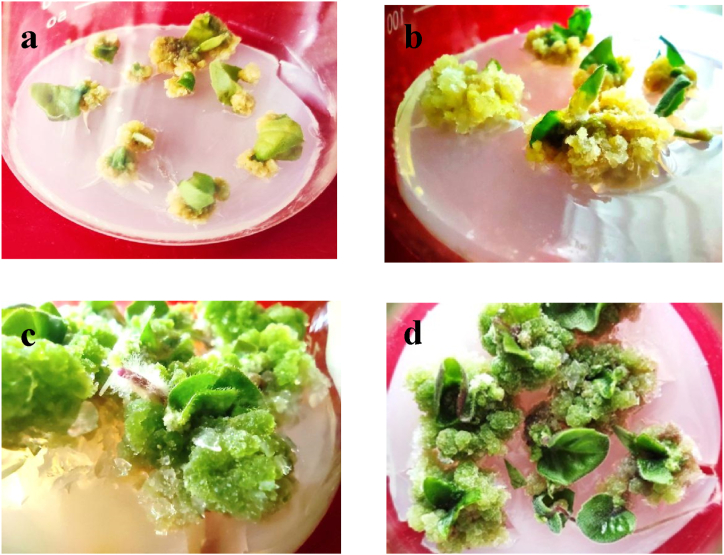
(a–d). Matured calli produced on MS media supplemented with (a) 4.0 mg/L 2,4-D & 1.0 mg/L BAP, (b) 3.0 mg/L 2,4-D & 2.0 mg/l BAP, (c) 2.0 mg/L BAP & 2.0 mg/L NAA, (d) 4.0 mg/L BAP & 4.0 mg/L NAA.

**Fig. 3:**
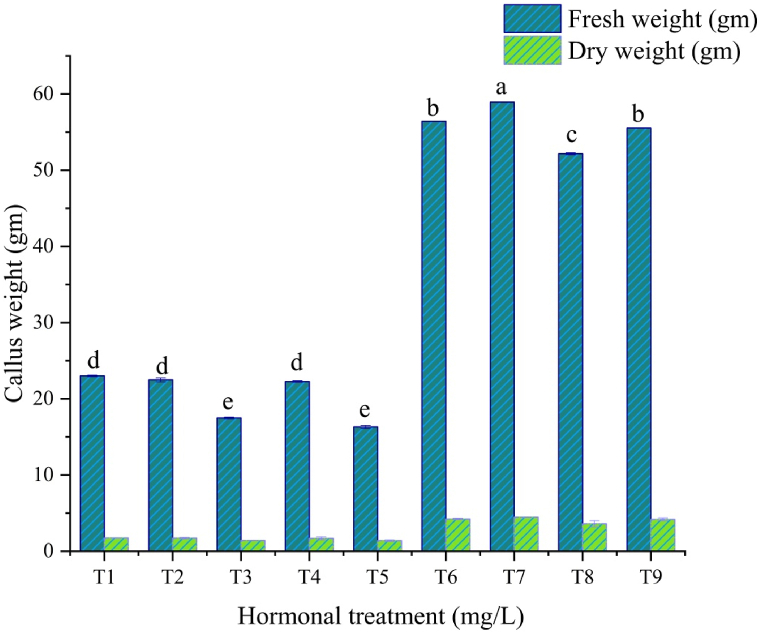
Fresh and dry weights of 30 calli of each combination of MS with 2,4-D: BAP (mg/L); T1 (3:2); T2 (2:2); T3 (4:2); T4 (2:1); T5 (4:1), BAP with NAA (mg/L); T6 (1:2); T7 (2:2); T8 (2:4); T9 (4:4). Values are represented as mean ± SD, where n = 5. The ranking is done as per Tukey's test at the p < 0.05 level of significance.

The explants were inoculated in MS media supplemented with different combinations and concentrations of PGRs. It was observed that in all combinations, the explants responded to 100 % callus induction within 14–18 days of inoculation [Fig. 2 (a – d)]. It was noticed that green calli showed up on all BAP and NAA media combinations. Furthermore, the friable and soft light brown color of the calli was produced on MS with different concentrations and combinations of 2,4-D and BAP. Among the various concentrations of applied phytohormones for callus induction from regenerated leaf explants, BAP (2.0 mg/L) and NAA (2.0 mg/L) induced calli with the highest fresh and dry weights (Fig. 3).

### 3.3 Antibacterial activity

Different *in vivo* and *in vitro* plant material extracts of *G. procumbens* exhibited varying antibacterial activity against tested microorganisms, as recorded in Table 2. The field-grown leaf extract and the regenerated leaf extract showed antibacterial activity against all tested bacteria, whereas the callus extract did not show any antibacterial properties against *P. aeruginosa*. Regenerated stem extract exhibited no antibacterial properties against any bacteria in three replications. A satisfactory result was obtained with regenerated leaf extract, showing the highest inhibitory zone (24 ± 0.00 mm) against *P. aeruginosa*, where doxycycline was a positive control (Table 2). With a callus extract concentration of 2.5 mg/mL, the lowest inhibition zone (8.980.05 mm) was found against *B. megaterium* (Table 2). A statistically significant interaction between the effects of the extract and the type of bacteria on the inhibition zone was detected by a two-way ANOVA (Table 3).

**Table 2:** Antibacterial activity of *G. procumbens* field leaf extract, callus extract, *in vitro* regenerated leaf extract and stem extract.

Bacterial strain	Extracts	Antibiotic	Zone of inhibition for			
			antibiotic (mm)	2.5 mg/mL (mm)	5 mg/mL (mm)	10 mg/mL (mm)
*E. coli*	Field Leaf	AM 10	10.88 ± 0.6	16.5 ± 0.6	19.75 ± 0.5	20 ± 0.00
		DO 30	30 ± 0.00	15.05 ± 0.1	16.25 ± 0.5	17 ± 0.4
	Callus	AM 10	10.88 ± 0.25	16 ± 0.00	17 ± 0.00	20 ± 0.00
		DO 30	30 ± 0.00	13 ± 0.00	14 ± 0.00	16 ± 0.00
	Regenerated Leaf	AM 10	10.88 ± 0.6	19 ± 0.00	19.25 ± 0.3	21.25 ± 0.5
		DO 30	28.50 ± 1.0	17 ± 0.00	18.75 ± 1	20 ± 0.00
	Regenerated Stem	AM 10	**-**	**-**	**-**	**-**
		DO 30	**-**	**-**	**-**	**-**
*S. typhimurium*	Field Leaf	AM 10	10.25 ± 0.4	16.5 ± 0.6	17.38 ± 0.5	17 ± 1.4
		DO 30	30 ± 0.00	11.25 ± 1.5	11.25 ± 0.5	14 ± 2.4
	Callus	AM 10	10.25 ± 0.5	15.5 ± 0.6	16.75 ± 1	17 ± 0.00
		DO 30	30 ± 0.00	15.5 ± 0.6	16.25 ± 0.5	16.88 ± 0.25
	Regenerated Leaf	AM 10	10.25 ± 1.2	15 ± 1.8	15.25 ± 0.5	19.5 ± 0.6
		DO 30	25 ± 0.00	15 ± 0.00	18 ± 0.00	18 ± 0.00
	Regenerated Stem	AM 10	**-**	**-**	**-**	**-**
		DO 30	**-**	**-**	**-**	**-**
*P. aeruginosa*	Field Leaf	AM 10	10.5 ± 0.6	10.25 ± 0.5	12 ± 0.00	13.88 ± 0.25
		DO 30	16.5 ± 0.00	16.75 ± 0.5	17 ± 0.8	16.5 ± 0.6
	Callus	AM 10	10.5 ± 0.6	**-**	**-**	**-**
		DO 30	16 ± 0.00	**-**	**-**	**-**
	Regenerated Leaf	AM 10	10.50 ± 0.00	14 ± 0.00	16.50 ± 0.00	18.50 ± 0.00
		DO 30	16.50 ± 0.00	19.50 ± 0.00	22.80 ± 0.00	24 ± 0.00
	Regenerated Stem	AM 10	**-**	**-**	**-**	**-**
		DO 30	**-**	**-**	**-**	**-**
*B. cereus*	Field Leaf	AM 10	10 ± 0.00	16 ± 0.8	14.5 ± 1.7	16.25 ± 1
		DO 30	10 ± 0.00	15.5 ± 0.6	14.5 ± 1	16 ± 0.00
	Callus	AM 10	10 ± 1.4	16.13 ± 0.25	16.25 ± 0.3	17 ± 0.00
		DO 30	11 ± 0.00	14.63 ± 0.75	14.5 ± 0.4	16 ± 0.00
	Regenerated Leaf	AM 10	10 ± 0.00	19 ± 0.00	20.25 ± 0.00	22 ± 0.00
		DO 30	11 ± 0.00	18 ± 0.00	20.5 ± 0.00	23 ± 0.00
	Regenerated Stem	AM 10	**-**	**-**	**-**	**-**
		DO 30	**-**	**-**	**-**	**-**
*B. megaterium*	Field Leaf	AM 10	8.5 ± 1	**-**	12.13 ± 0.25	13.38 ± 0.5
		DO 30	30 ± 0.00	13.25 ± 1	13.13 ± 0.6	13.13 ± 0.7
	Callus	AM 10	8.5 ± 0.9	8.98 ± 0.05	12.5 ± 0.7	13.88 ± 0.25
		DO 30	29.5 ± 1.00	12.25 ± 0.6	13.5 ± 1.3	13.88 ± 0.25
	Regenerated Leaf	AM 10	9 ± 0.00	12 ± 0.00	13 ± 0.00	14 ± 0.00
		DO 30	30 ± 0.00	16 ± 0.00	18.25 ± 0.00	21 ± 0.00
	Regenerated Stem	AM 10	**-**	**-**	**-**	**-**
		DO 30	**-**	**-**	**-**	**-**

Values are represented as mean ± SD where n = 3. AM = Ampicillin, DO = Doxycycline.

**Table 3:** Result of two-way ANOVA for plant part extracts and bacteria.

Source	Sum of Squares	df	Mean Square	F	*p*–value
Type of Extract	512.85	2	256.43	9.84	<0.001
Type of Bacteria	611.78	4	152.95	5.87	<0.001
Type of Extract * Type of Bacteria	619.54	8	77.44	2.97	0.005
Error	2735.24	105	26.05		
Total	32928.45	120			

A simple principal effect analysis was done because the interaction effect between the type of extract and the type of bacteria was significant (*p* = 0.005). Table 4 displays the estimated marginal mean and its 95 % confidence intervals.

**Table 4:** Estimated Marginal Mean from the simple main effect.

Type of Extract	Types of Bacteria	Mean	SE	95 % CI	
				Lower Bound	Upper Bound
Field leaf extract (1)	*E. coli*	18.01	1.81	14.43	21.58
	*S. typhimurium*	16.27	1.81	12.69	19.84
	*P. aeruginosa*	14.14	1.81	10.56	17.72
	*B. cereus*	14.08	1.81	10.50	17.66
	*B. megaterium*	12.94	1.81	9.36	16.52
Callus extract (2)	*E. coli*	15.70	1.81	12.13	19.28
	*S. typhimurium*	17.83	1.81	14.25	21.41
	*P. aeruginosa*	3.31	1.81	**−**0.27	6.89
	*B. cereus*	14.19	1.81	10.61	17.77
	*B. megaterium*	14.14	1.81	10.56	17.72
Regenerated leaf extract (3)	*E. coli*	20.53	1.81	16.95	24.11
	*S. typhimurium*	18.03	1.81	14.45	21.61
	*P. aeruginosa*	17.79	1.81	14.21	21.37
	*B. cereus*	17.97	1.81	14.39	21.55
	*B. megaterium*	16.03	1.81	12.45	19.61

Using the LSD method, a Post Hoc pairwise multiple comparisons test was done to see where the differences between the types of extract and types of bacteria were. Table 5 displays the mean differences based on the estimated marginal means. According to the LSD results, extract 2 (callus extract) significantly inhibited *P. aeruginosa* with a smaller zone of inhibition than extract 1 (field leaf extract) (*p* < 0.001). Similar to extract 2, extract 3 (regenerated leaf extract) significantly increased the zone of inhibition against *P. aeruginosa* compared to extract 2 (*p* < 0.001). However, there was no real distinction between extracts 3 and 1 (*p* = 0.156). Other bacteria were also subjected to pairwise comparisons, but no distinctive differences were revealed.

**Table 5:** The LSD pairwise comparisons test.

Types of Bacteria	Type of Extract^a^ (A)	Type of Extract (B)	Mean Difference (A-B)	SE	*p*–value	95 % CI for Difference	
						Lower Bound	Upper Bound
*E. coli*	1	2	2.30	2.55	0.369	**−**2.76	7.36
	2	3	**−**4.83	2.55	0.061	**−**9.89	0.23
	3	1	2.53	2.55	0.325	**−**2.54	7.59
*S. typhimurium*	1	2	**−**1.56	2.55	0.542	**−**6.62	3.50
	2	3	**−**0.20	2.55	0.937	**−**5.26	4.86
	3	1	1.77	2.55	0.491	**−**3.30	6.83
*P. aeruginosa*	1	2	10.83*	2.55	<0.001	5.77	15.89
	2	3	**−**14.48*	2.55	<0.001	**−**19.54	**−**9.42
	3	1	3.65	2.55	0.156	**−**1.41	8.71
*B. cereus*	1	2	**−**0.11	2.55	0.966	**−**5.17	4.95
	2	3	**−**3.78	2.55	0.142	**−**8.84	1.28
	3	1	3.89	2.55	0.131	**−**1.17	8.95
*B. megaterium*	1	2	**−**1.20	2.55	0.639	**−**6.26	3.86
	2	3	**−**1.89	2.55	0.460	**−**6.95	3.17
	3	1	3.09	2.55	0.228	**−**1.97	8.15

*The mean difference is significant at the 5 % level.

a Type of extract: 1 = field leaf extract; 2 = callus extract and 3 = regenerated leaf extract.

**Fig. 4:**
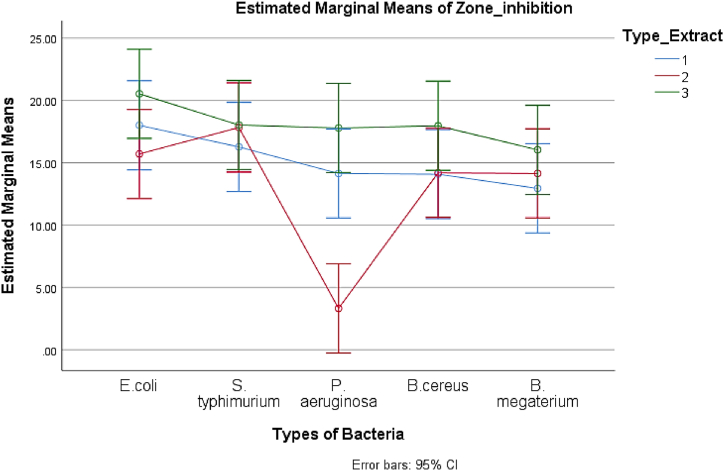
Profile plot showing effect of extracts on different types of bacteria.

A profile plot (shown in Fig. 4) was made based on estimated marginal means to show how the extracts, bacteria, and inhibition zones work together. The interaction demonstrated in the profile plot was that the extract 3 (regenerated leaf extract) line demonstrated the highest antibacterial activity. In contrast, the extract 1 (field leaf extract) line exhibited unusual activity, but the extract 2 (callus extract) line displayed significant antibacterial activity fluctuation. The significant interaction between the type of extracts and the bacteria was determined by the crossing of extract line 2 with extract lines 1 and 3 (Fig. 4).

### 3.4 Determination of minimum inhibitory concentrations (MIC's) & minimum bactericidal concentration (MBC)

The field leaf, regenerated leaf, and callus methanolic extracts showed the most significant antimicrobial activity among the plant extracts tested, both *in vivo* and *in vitro*. So, the MIC values of these extracts were measured, and Table 6 shows the results. Except for the interaction between callus extract and *P. aeruginosa*, the MIC values of all the extracts were interesting because the bacteriostatic activity started at a low concentration (2–4 mg/mL). Therefore, to inhibit *P. aeruginosa*, a higher concentration of callus extract was necessary.

**Table 6:** MIC and MBC's of the effective *in vivo* and *in vitro* plant materials extract.

Tested bacteria	Different extracts	Bacterial growth in Muller-Hinton medium (Plant materials extracts concentrations) (mg/mL)																	MIC mg/mL	MBC mg/mL
		0.1	1	2	3	4	5	6	7	8	9	10	11	12	13	14	15	16		
*E. coli*	L	+	+	**-**	**-**	**-**	**-**	**-**	**-**	**-**	**-**	**-**	**-**	**-**	**-**	**-**	**-**	**-**	2	3
	C	+	+	+	**-**	**-**	**-**	**-**	**-**	**-**	**-**	**-**	**-**	**-**	**-**	**-**	**-**	**-**	3	4
	RL	+	**-**	**-**	**-**	**-**	**-**	**-**	**-**	**-**	**-**	**-**	**-**	**-**	**-**	**-**	**-**	**-**	1	2
*S. typhimurium*	L	+	+	**-**	**-**	**-**	**-**	**-**	**-**	**-**	**-**	**-**	**-**	**-**	**-**	**-**	**-**	**-**	2	3
	C	+	+	+	**-**	**-**	**-**	**-**	**-**	**-**	**-**	**-**	**-**	**-**	**-**	**-**	**-**	**-**	3	4
	RL	+	+	+	**-**	**-**	**-**	**-**	**-**	**-**	**-**	**-**	**-**	**-**	**-**	**-**	**-**	**-**	2	3
*P. aeruginosa*	L	+	+	**-**	**-**	**-**	**-**	**-**	**-**	**-**	**-**	**-**	**-**	**-**	**-**	**-**	**-**	**-**	2	3
	C	+	+	+	**-**	**-**	**-**	**-**	**-**	**-**	**-**	**-**	**-**	**-**	**-**	**-**	**-**	**-**	3	4
	RL	+	+	+	**-**	**-**	**-**	**-**	**-**	**-**	**-**	**-**	**-**	**-**	**-**	**-**	**-**	**-**	2	3
*B. cereus*	L	+	+	+	**-**	**-**	**-**	**-**	**-**	**-**	**-**	**-**	**-**	**-**	**-**	**-**	**-**	**-**	3	4
	C	+	+	+	**-**	**-**	**-**	**-**	**-**	**-**	**-**	**-**	**-**	**-**	**-**	**-**	**-**	**-**	3	4
	RL	+	+	**-**	**-**	**-**	**-**	**-**	**-**	**-**	**-**	**-**	**-**	**-**	**-**	**-**	**-**	**-**	2	3
*B. megaterium*	L	+	+	+	+	**-**	**-**	**-**	**-**	**-**	**-**	**-**	**-**	**-**	**-**	**-**	**-**	**-**	4	5
	C	+	+	**-**	**-**	**-**	**-**	**-**	**-**	**-**	**-**	**-**	**-**	**-**	**-**	**-**	**-**	**-**	2	3
	RL	+	+	**-**	**-**	**-**	**-**	**-**	**-**	**-**	**-**	**-**	**-**	**-**	**-**	**-**	**-**	**-**	2	3

### 3.5 Brine shrimp lethality test

**Fig. 5:**
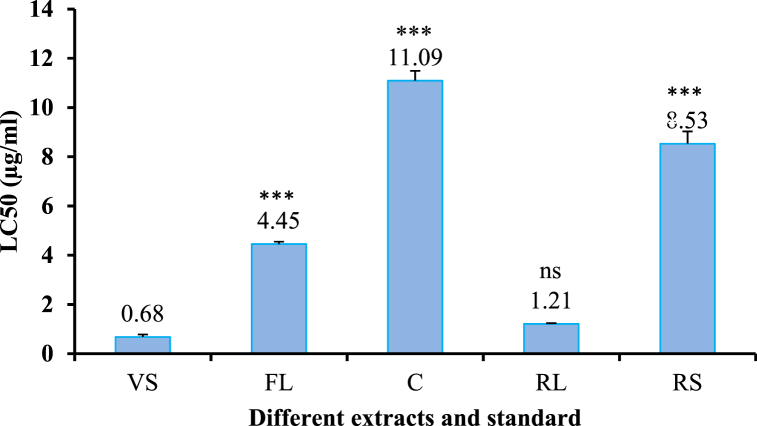
Values of the LC50 for different types of extracts and standards. VS = Vincristine sulfate, FL = Field leaf extract, C = Callus extract, RL = Regenerated leaf extract, and RS = Regenerated stem extract. Values are presented as Mean ± SD with n = 3. Degrees of significance determined using an ANOVA with Post Hoc Tukey's test at a 5 % significance level to compare the extracts with the vehicle are *** = highly significant and ns = no significant difference.

Fig. 5 displays the LC50 values for both *in vivo* and *in vitro* plant part extracts that were calculated using linear regression analysis. In comparison to the standard, it is clear that callus extract is the least toxic, and regenerated leaf extract is the most toxic, with the lowest LC50 value.

### 3.6 *In vitro* cytotoxicity test

**Fig. 6:**
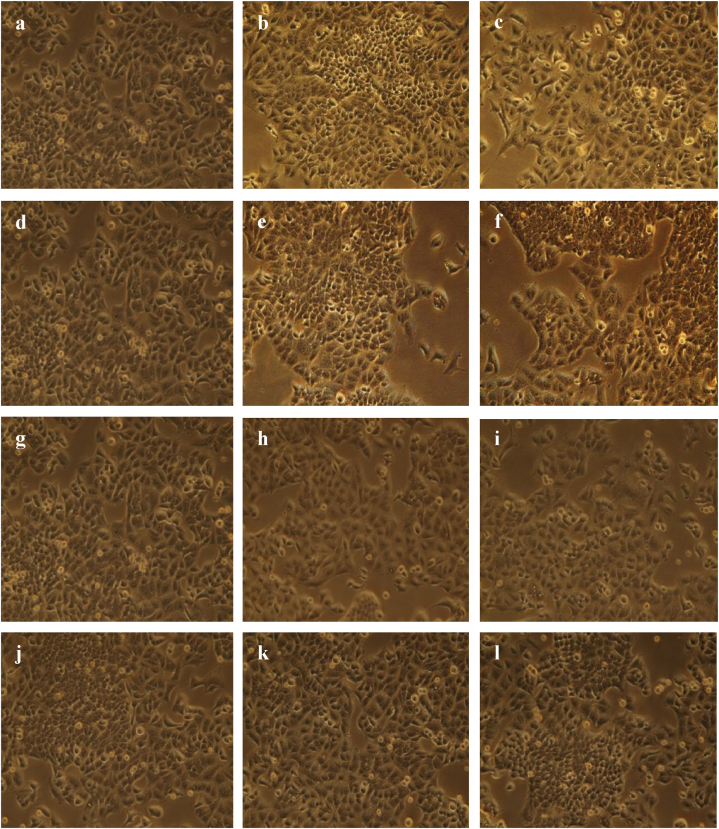
(a–l). Microscopic (Olympus inverted microscope CKX41) images (20 × ) of the cytotoxic activity of different plant extracts of *G. procumbens* determined on a human lung cancer cell line (A549, CSL, Germany) at different concentrations after 24 h. (a) Control for field leaf extract (b) field leaf extract 10 μg/mL (c) field leaf extract 10 μg/mL (d) control for callus extract (e) callus extract 10 μg/mL (f) callus extract 20 μg/mL (g) control for regenerated leaf extract (h) regenerated leaf extract 10 μg/mL (i) regenerated leaf extract 20 μg/mL (j) control for regenerated stem extract (k) regenerated stem extract 10 μg/mL (l) regenerated stem extract 20 μg/mL.

**Fig. 7:**
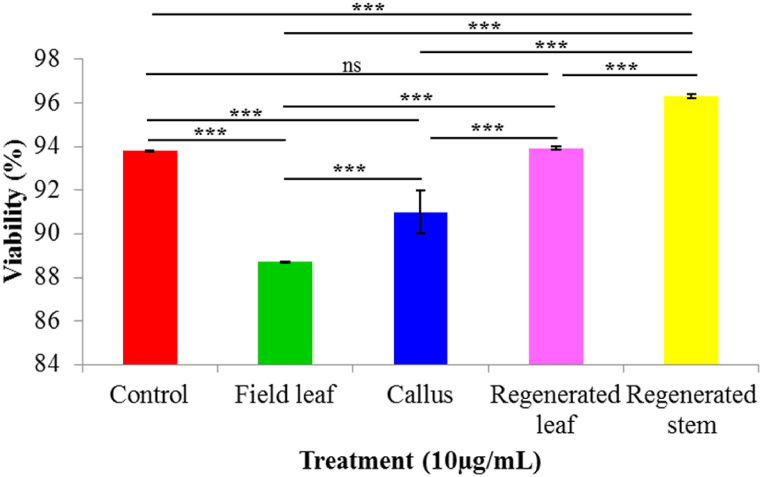
Comparison of the cytotoxic activities of different extracts at a concentration of 10 μg/mL and a control. All data are presented as mean ± SD with n = 3. The significance of mean differences is determined using a one-way ANOVA with Post Hoc Tukey's test at the 5 % and 1 % significance levels. *** = highly significant, ** = significant, ns = not significant.

**Fig. 8:**
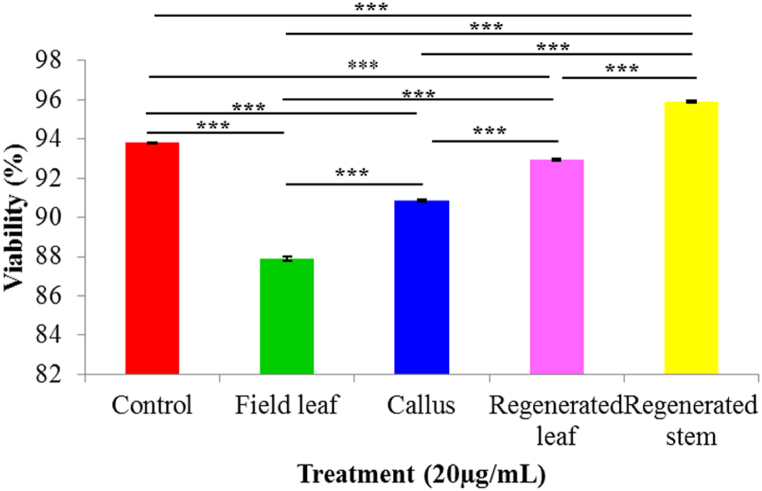
Comparison of cytotoxic activity of different extracts at concentration 20 μg/mL and control. All data are presented as mean ± SD with n = 3. The significance of mean differences is determined using a one-way ANOVA with Post Hoc Tukey's test at 5 % and 1 % significance levels. *** = highly significant, ** = significant, ns = not significant.

After 24 h of exposure, the human lung cancer cell line was tested for the ability of plant part extracts to be cytotoxic. A dose-dependent reduction in cell viability was seen [Fig. 6 (a - l)]. Cell viability was 88.70 % with 10 μg/mL field leaf extract and declined to 87.90 % with 20 μg/mL field leaf extract. When cells were incubated with 10 μg/mL and 20 μg/mL callus extract, the percentages of cell viability were 91.0 and 89.90, respectively. The percentage of cell viability decreased with increasing concentration compared to the control, indicating that both the field leaf extract and the callus extract inhibited cell proliferation. The percentage of cell viability slightly increased (93.80 %) when treated with regenerated leaf extract compared to the control but again decreased (92.90 %) at higher concentrations. Regenerated stem extract produced 96.30 % cell viability at a concentration of 10 μg/mL and 95.90 % at a concentration of 20 μg/mL. The increase in cell viability (%) after treatment with regenerated leaf and stem extracts suggests that these extracts can induce cell growth (Fig. 7, Fig. 8).

**Fig. 9:**
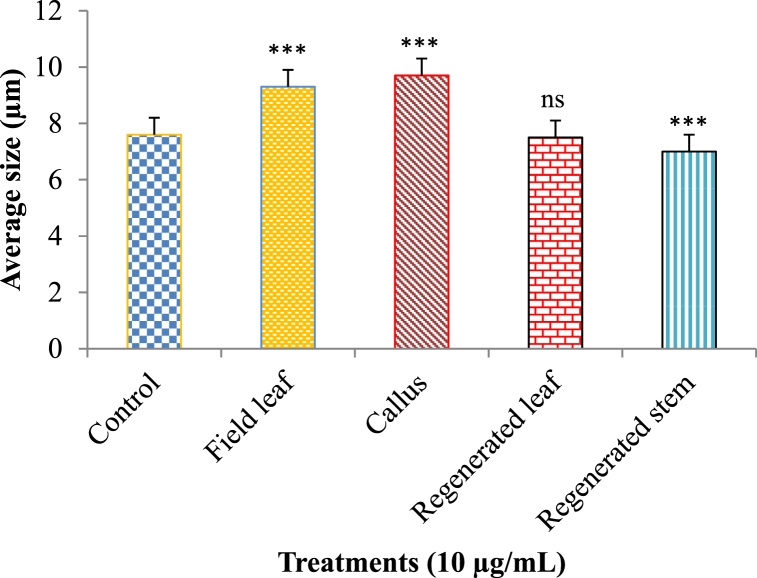
Variation of average sizes (μm) of the cultured human lung cancer cells treated with control and different extracts (10 μg/mL). The results are expressed as the mean ± SD, with n = 3. The degrees of significance calculated by employing ANOVA with Post Hoc Tukey's test were *** = significant at the 1 % level, ** = significant at the 5 % level, and ns = not significant.

**Fig. 10:**
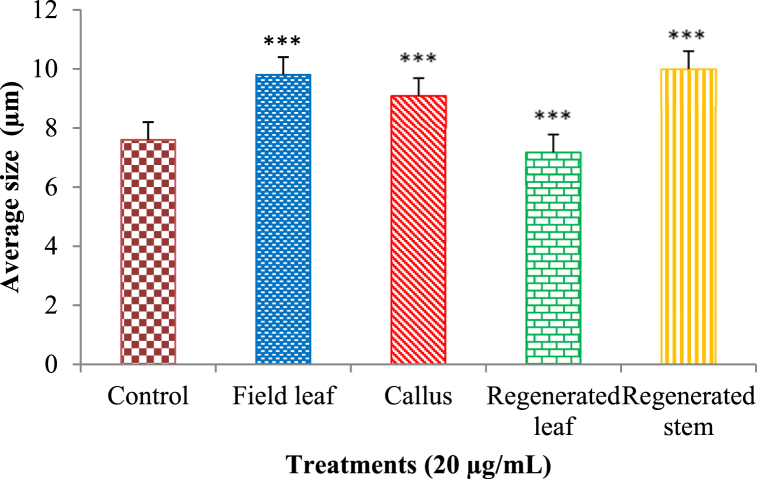
Variation of average sizes (μm) of the cultured lung cancer cells treated with control and different extracts (20 μg/mL). The results are expressed as the mean ± SD, with n = 3. The degrees of significance calculated by employing ANOVA with Post Hoc Tukey's test were *** = significant at the 1 % level, ** = significant at the 5 % level, and ns = not significant.

Quite the opposite was seen when looking at cellular size. Compared to the control group (7.60 μm), cells treated with 10 μg/mL field leaf and callus extract had larger average sizes (μm) of 9.30 and 9.70, respectively. The regenerated leaf extract of 10 μg/mL concentration helped to decrease the size of the cells to 7.50 μm, and the regenerated stem extract did the same to obtain a cell concentration of 7.0 μm (Fig. 9). Cells were reduced to 7.50 μm in size with the aid of a 10 μg/mL concentration of regenerated leaf extract, and to 7.00 μm with the aid of a 10 μg/mL concentration of regenerated stem extract (Fig. 9). In response to a higher concentration of field leaf extract (20 μg/mL), the cell size also increased, reaching 9.8 μm. In contrast, the cell size shrank to 9.10 μm when treated with 20 μg/mL callus extract compared to the size at the control and lower callus extract doses. Cell size was also reduced after treatment with the extract of regenerated leaves, which was 7.20 μm. Cells grew to 10.0 μm in diameter after being exposed to 20 μg/mL of regenerated stem extract (Fig. 10). To fully understand whether or not these extracts have any anticancer properties, more targeted research is required.

The experiment results suggest that field leaf extract has more pronounced anti-cell proliferative properties than callus extract, suggesting it is more effective at preventing the growth of abnormal cells. This result suggests that an extract from the field leaf and callus of *G. procumbens* could be a source of new anti-cancer drugs. It happened because the extracts significantly slowed cell growth. In contrast, the regenerated leaf and regenerated stem extracts possess cell-inducible properties. Therefore, screening bioactive compounds from these extracts can verify these properties. Subsequently, further investigation can be conducted to analyze and identify these bioactive compounds, as they are primarily responsible for the induction of cell growth or the inhibition of cell proliferation.

## 4 Discussion

*In vitro* regeneration is an effective technique for producing disease-free and genetically accurate plants [14]. However, as numerous factors, such as the type of explants, combination of plant growth regulators on media, culturing parameters, genotype, etc., influence the *in vitro* regeneration of plants, their biochemical properties may differ from those of *in vivo* materials [29,36,37].

MS media supplemented with 1.0 mg/l BAP and 0.5 mg/L IAA were the best for shoot regeneration from nodal explants in this study. This outcome was consistent with what Banu *et al*. (2017) had reported in their earlier investigation [38]. However, Azad and Amin (2017) found that nodal explants produced the highest number of shoots in MS with only 4.0 μM BAP [39], and the highest shoot proliferation was established on MS medium supplemented with 4.0 μM BAP and 2.0 NAA using leaf explants [5]. A medicinal plant called *Dolichandra unguis-cati* (L.) Lohmann showed the best morphogenetic response on MS medium with 0.5 mg/L BAP, and 1.0 mg/L TDZ added in terms of explant response, number of shoots, and length of shoots [40]. Many other medicinal species have found that full strength of MS medium works best for axillary shoot proliferation, such as *G. porcumbens* [41,42]; *Aspilia africana* [43]; *Phaleria macrocarpa* [44].

For callus induction, cultured plant leaves from regenerated plants were used. Depending on the callus morphology, fresh weight, and dry weight, the MS with a 2.0 mg/L BAP and 2.0 mg/L NAA combination was considered the best in the current study. In a previous study, the combination of 0.1 mg/L 2, 4-D, and 0.1 mg/L BAP was the most effective for callus induction from leaf explants [45]. Banu *et al*. (2017) reported that *G. procumbens* leaf explants showed the best callogenic response when cultured in MS media supplemented with 2.0 mg/L BAP, 1.0 mg/L IAA, and 0.5 mg/L Kn [38]. Similar findings were achieved using the same PGRs (2.2 μM BAP + 2.7 μM NAA) for another important medicinal plant of the Asteraceae family, safflower (*Carthamus tinctorius* L.), via callus-mediated organogenesis [46]. So, this regeneration protocol is reliable and more cost-effective than others.

Plants are a significant source of essential drugs beneficial to the well-being of individuals. People frequently utilize medicinal plants to treat and prevent dangerous diseases [47]. As the number of resistant pathogens rises, the use of medicinal plants for their antimicrobial properties is gaining popularity [48]. Some plant species have abundant sources of biomaterials with a variety of biological activities, including antibacterial, antifungal, and antioxidant properties [11,49]. In this study, 80 % methanolic extracts of field leaf, regenerated leaf, and callus from *G. procumbens* demonstrated antibacterial activity against all bacteria tested. However, according to a previous report, the crude methanolic extract of *G. procumbens* leaves exhibited no antibacterial activity [50]. All of the extracts in the current study demonstrated inhibitory activity at very low concentrations, but the outcome is consistent with ours in terms of extract type. Methanolic extract of *G. procumbens* leaves demonstrated the highest antimicrobial activity against *S. aureus* at 400 mg/mL concentrations with a 10.5 mm inhibition zone [51]. In addition, the regenerated leaf extract in this study demonstrated the highest antibacterial potential.

The regenerated leaf extract of *G. procumbens* was the most toxic (LC50 = 1.21 μg/mL) in a bioassay measuring the lethality of brine shrimp. The LC50 value obtained for field leaf was 4.45 μg/mL, indicating a high level of toxicity. The MeOH–H_2_O extract of *G. procumbens* leaves was found to have significant cytotoxic activity with a reported LC50 value of 3.98 μg/mL [52]. Compared to the standard, the callus extract has the highest LC50 value (11.09 μg/mL) and is the least toxic in this study. This bioassay indicated that the extracts contained bioactive compounds.

Field leaf extract and callus extract were found to have anti-cell proliferation properties in the current investigation. In contrast, the regenerated leaf and stem extracts have cell-inducing properties. The screening of bioactive components from these extracts can confirm these properties. The antiproliferative activity of *G. procumbens* leaf extract against osteosarcoma cell lines has been reported [53]. Rats were used to test the efficacy of an ethanolic extract of *G. procumbens* leaves in preventing colon cancer [54]. It has also been reported that *G. procumbens* leaf extract inhibits breast cancer cell proliferation [55]. The traditional uses of *G. procumbens* for treating of leukemia, uterine, and breast cancers [56,57] prompted scientific investigation of its antitumor properties. To our knowledge, no report has yet described the *in vitro* cytotoxicity of *G. procumbens* regenerated plant material. There are numerous findings regarding the biological and therapeutic benefits of plant extracts. This research focused on the effects of both *in vitro* and *in vivo*-grown plant parts of *G. procumbens*.

## 5 Conclusion

We developed a protocol for the efficient and frequent regrowth of a medicinal plant, *G. procumbens*. According to a comparison of *in vivo* and *in vitro* plant parts, the antibacterial activity of the leaves of *G. procumbens* plants grown *in vitro* is also higher than that of the mother plant. Regenerated leaf extracts were the most effective against all of the tested bacteria, according to the MBC and MIC results. In addition, the regenerated leaf extract had the lowest LC50 value and was the most toxic substance in a bioassay. This tissue culture-generated plant population, which has a consistent genetic makeup, can, to a desirable extent, meet the rising local demand for traditional medicine, pharmaceutical firms, and drug discovery research. The findings of this study provide information on the biochemical characteristics of the *in vitro*-grown parts of the *G. procumbens* species. More specialized research is needed to find the bioactive compounds responsible for the biological activities that plant parts show when grown *in vitro*.

## Data availability statement

Data included in article/referenced in article.

## Ethics approval and consent to participate

Not applicable.

## CRediT authorship contribution statement

**Sanchita Saha:** Conceptualization, Data curation, Formal analysis, Methodology, Resources, Software, Validation, Visualization, Writing – original draft, Writing – review & editing. **G M Al Amin:** Conceptualization, Data curation, Formal analysis, Funding acquisition, Investigation, Methodology, Project administration, Resources, Software, Supervision, Validation, Visualization, Writing - original draft, Writing - review & editing. **Md Salim Khan:** Data curation, Formal analysis, Investigation, Methodology, Visualization. **Barna Goswami:** Data curation, Formal analysis, Investigation. **Farhana Afroz:** Data curation, Formal analysis, Investigation, Methodology. **Md Ahashan Habib:** Data curation, Formal analysis, Investigation. **Shahina Akter:** Data curation, Formal analysis, Investigation. **Tanjina Akhtar Banu:** Conceptualization, Data curation, Formal analysis, Investigation, Methodology, Supervision, Validation.

## Declaration of competing interest

The authors declare that they have no known competing financial interests or personal relationships that could have appeared to influence the work reported in this paper.

## Abbreviations

MS: Murashige and Skoog
2,4-D: 2,4-Dichlorophenoxyacetic acid
NAA: Napthalene acetic acid
BAP: 6-Benzylaminopurine
IBA: Indole butyric acid
PGR: Plant growth regulators
LSD: Least significant difference
MIC: Minimum inhibitory concentration
MBC: Minimum bactericidal concentration
DMSO: Dimethylsulphoxide
FBS: fetal bovine serum
HEPES: N-(2-Hydroxyethyl)piperazine-N′-(2-ethanesulfonic acid)
AM: Ampicillin
DO: Doxycycline
MHB: Mueller Hinton Broth
CLSI: Clinical and Laboratory Standards Institute
CFU: Colony-forming unit
LC50: Lethal concentration 50
SD: Strand deviation
